# Simulation and Structural Analysis of a Flexible Coupling Bionic Desorption Mechanism Based on the Engineering Discrete Element Method

**DOI:** 10.3390/biomimetics9040224

**Published:** 2024-04-08

**Authors:** Jinguang Li, Hongyan Qi, Yunhai Ma, Peng Gao, Baoguang Wu

**Affiliations:** 1 The College of Biological and Agricultural Engineering, Jilin University, 5988 Renmin Street, Changchun 130025, China; jgli18@mails.jlu.edu.cn (J.L.); qhy18@mails.jlu.edu.cn (H.Q.); penggao15@mails.jlu.edu.cn (P.G.); wubg@jlu.edu.cn (B.W.); 2 Key Laboratory of Bionic Engineering, Ministry of Education, Jilin University, 5988 Renmin Street, Changchun 130025, China

**Keywords:** sandfish (*Scincus scincus*), flexible desorption, stress concentration, wedged structure serrated structure, bionic non-smooth surface

## Abstract

Soil adhesion is one of the important factors affecting the working stability and quality of agricultural machinery. The application of bionic non-smooth surfaces provides a novel idea for soil anti-adhesion. The parameters of sandy loam with 21% moisture content were calibrated by the Engineering Discrete Element Method (EDEM). The final simulated soil repose angle was highly consistent with the measured soil repose angle, and the obtained regression equation of the soil repose angle provides a numerical reference for the parameter calibration of different soils. By simulating the sinusoidal swing of a sandfish, it was found that the contact interface shows the phenomenon of stress concentration and periodic change, which reflects the effectiveness of flexible desorption and soil anti-adhesion. The moving resistance of the wedge with different wedge angles and different serrated structures was simulated. Finally, it was found that a 40° wedge with a high-tail sparse staggered serrated structure on the surface has the best drag reduction effect, and the drag reduction is about 10.73%.

## 1. Introduction

There are many problems when the soil-engaging components of ground machinery contact the soil. An example is soil adhesion, which makes it very easy to form extensive soil nuclei at the surface. Soil nuclei not only affect the working quality and efficiency of the machinery but also increase the working resistance and energy consumption [1,2]. When ground machinery is in contact with the soil, the soil easily adheres to the surface of the machinery due to the humidity and viscosity of the soil. In turn, a large adhesion force is generated, which makes it more difficult for the components to operate smoothly. The increase in mechanical working resistance caused by soil adhesion directly affects the energy consumption and working efficiency of machinery, such as disc harrows, furrow plows, subsoilers, rotary cultivators, and excavators [3,4]. When the soil adhesion is large, it can directly determine whether the machine is suitable for operation.

In recent years, many scholars have carried out a series of studies and put forward many theories on the anti-adhesion and desorption of agricultural machinery. An example is the development of material science [5] in the applications of anti-adhesion, desorption, and wear resistance. Vibration drag reduction [6,7] and electro-osmotic drag reduction [8] also have good effects in reducing adhesion and desorption. In addition, the development of bionics provides a new idea for reducing the adhesion and desorption of soil-engaging components. The theory of profiling design and non-smooth surface drag reduction has been rapidly developed and applied and also has been widely recognized [9,10]. The non-smooth surface structure also has a good anti-adhesion and desorption effect on the soil-engaging components of agricultural machinery, such as the structure of convex hulls, pits, and ribs [11,12]. Numerous studies have shown that the shape, depth, and density of surface textures have a significant impact on friction, but under different working conditions such as pressure and load, they can affect the effect of a surface structure and even lead to its failure [13,14,15,16].

After long-term underground life and environmental adaptation, the morphological structure and surface microstructure of soil animals have undergone adaptive changes, which are perfectly suitable for underground life. Underground excavation and walking animals such as pangolins and moles [17,18] have unique body structures and surface microstructures that help them advance in the soil with less resistance. Studies have found that the body surfaces of some reptiles such as snakes and lizards are composed of multi-level structures, showing a multi-dimensional flexible layering, the structure of which has a good desorption function [19,20].

The sandfish (*Scincus scincus*) can rely on the swinging of its body rather than its limbs to move freely in the sand, which is the reason for its name [21]. The perfect head wedge structure of the sandfish can make it enter the soil with the minimum resistance in the movement. The body swing mode [22,23] can obtain the maximum forward power during the movement, and the multi-dimensional scale structure on the body surface plays a good desorption effect. However, most research mainly focuses on the anti-adhesion and desorption of the scale material properties [24,25,26,27,28] and the material push of the S-shaped movement mode [29,30] of the sandfish. Combined with the traditional soil dynamics and soil adhesion mechanism, there is little theoretical analysis and effective realization of soil desorption.

In this study, a three-dimensional scanner was used to obtain point cloud data of the head contour of the sandfish, and a reverse operation was carried out by Geomagic Studio software 12.0.0 to restore the three-dimensional structure of the sandfish head. Finally, some two-sided wedge models with different angles were constructed. The dorsal scale arrangement and the scale microstructure were observed and measured by a stereomicroscope and scanning electron microscope (SEM), and then some wedges with different structures on the surface were established. Through Engineering Discrete Element Method (EDEM) simulation, the stress distribution on the body surface of the sandfish model was analyzed, and the multi-dimensional flexible desorption mechanism of its body surface was explained. By coupling the flexible desorption mechanism of the swing body with the drag reduction mechanism of the head wedge structure, non-smooth surface wedge models were established. Then, the working resistance of the models in sandy loam was simulated and analyzed by the EDEM, and the models with serrated structures had an effective desorption drag reduction effect.

## 2. Materials and Methods

### 2.1. Parameter Acquisition of Bionic Prototype

#### 2.1.1. Body Motion Curve Acquisition and Model Establishment

The sandfish is predominantly distributed in arid and semi-arid regions of Africa. This lizard has the ability to swiftly burrow into the sand when faced with danger, relying solely on its swinging body in the sand. It can reach speeds of up to 0.3 m/s. In order to observe its burrowing behavior, we created and simulated a simulated habitat that mimicked its natural environment. Utilizing the body swing curve equation for the sandfish found in the existing literature, we constructed a swing curve model using CATIA V5R20 software.

#### 2.1.2. Head Contour Acquisition and Model Establishment

To obtain the head contour parameters of the sandfish, a sample of the head was first obtained. The specific procedure was as follows: adult sandfish were selected, anesthetized with ether, separated the head, back skin, abdominal skin, and other organs, fixed with methanol, dehydrated with different concentrations of alcohol, dried by freezing, and so on. A layer of matte white imaging agent was sprayed on the sandfish head specimen from all directions. The three-dimensional contour was then scanned using a three-dimensional scanner. The point cloud data obtained were processed through reverse engineering to generate a three-dimensional model. Finally, the wedge structure parameters of the model were obtained. Using CATIA V5R20, wedges with different angles were constructed.

#### 2.1.3. Microstructure Parameter Acquisition and Model Establishment

The dorsal epidermis samples of the sandfish were carefully chosen for analysis. The shape and arrangement of the epidermal scales were observed using a stereomicroscope. The serrated structural parameters were observed using an SEM, and the proportions were estimated concurrently. Wedge models with non-smooth surface structures were constructed using CATIA V5R20.

### 2.2. Simulation of EDEM

#### 2.2.1. Measurement of Soil Parameters

To improve the accuracy of EDEM, it is necessary to calibrate soil parameters in advance, such as soil texture, soil density, etc. In this study, the parameters required for simulation were calibrated by soil repose angle. When measuring the soil angle of repose, the soil of the local field tillage layer was taken, the soil texture was analyzed by the Laser Particle Size Measurement (BT-9300ST) made by Dandong Bettersize Instruments Ltd of China, and then the soil angle of repose was measured by soil accumulation.

#### 2.2.2. Calibration of Soil Simulation Parameters

When using the EDEM to simulate the interaction between soil and soil-engaging components, it primarily involves two types of material properties: soil particle properties and soil-engaging component properties. Soil-engaging components are typically made of metal materials, with this study specifically focusing on the use of 65 Mn steel. The key material properties of soil-engaging components include the metal’s Poisson ratio, density, and shear modulus. On the other hand, the principal material properties of soil particles consist of the particle’s Poisson ratio, density, and shear modulus. Additionally, other factors to consider in the simulation include the restitution coefficient, static friction coefficient, rolling friction coefficient between soil particles, as well as the physical model governing the interaction between soil particles and soil-engaging components.

When calibrating soil parameters using the EDEM software 2018, Design-Expert 12 was employed to optimize the simulation scheme. This decision was made due to the complexity of the numerous factors involved in the calibration process. The design parameters were optimized with the soil repose angle serving as the standard benchmark. The factors that made a significant contribution were identified through the Plackett–Burman and the steepest climbing experiment. Subsequently, a regression equation and a model were developed to optimize the angle of repose. This led to the determination of the optimal EDEM simulation parameters for sandy loam soil.

#### 2.2.3. Simulation and Analysis of Sandfish Body Swing Mode

The EDEM software 2018 was utilized to simulate and analyze the sandfish body model. Soil parameters, which had been calibrated, were input into EDEM to simulate the oscillatory motion of the sandfish body. This simulation enabled the examination of stress distribution on the model’s surface during movement. The theoretical mechanism of flexible desorption was also expounded upon.

#### 2.2.4. Simulation and Analysis of Wedge Structure and Serrated Structure

The head contour of the sandfish was acquired using a three-dimensional scanner. Subsequently, the point cloud data of the head contour was reconstructed through reverse engineering. Finally, the reconstructed data were imported into Microsoft Office Excel 2016 for curve fitting and angle measurement. Based on the obtained wedge angle, models with various wedge structures were created using CATIA V5R20. These wedges were then introduced into EDEM to simulate resistance, and the angle of the wedges was determined when the resistance was minimized. 

The study utilized scanning electron microscopy (SEM) to observe the serrated structure of lizard scales and measure the parameters of this structure. Subsequently, these parameters were scaled up and used to construct various non-smooth surface wedge models using CATIA V5R20 software. By simulating the penetration resistance of these different non-smooth surface wedges and comparing them to a smooth surface wedge, the optimal combination of wedge angle and surface microstructure interaction was determined.

## 3. Results

### 3.1. Bionic Prototype Structure Organization

**Figure 1:**
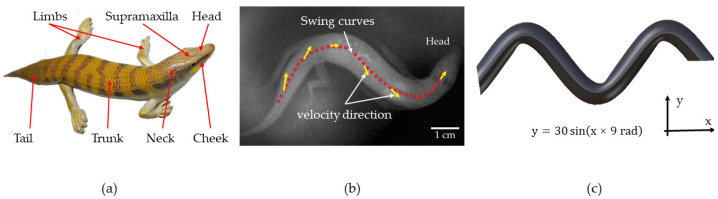
(**a**) Adult sandfish (*Scincus scincus*); (**b**) body shape when sandfish swing [21]; (**c**) model of sandfish body swing.

As illustrated in Figure 1a, an adult sandfish typically measures between 15 and 20 cm in body length, consisting of a head, neck, trunk, limbs, and tail. The entire body is covered in scaly tissue, which serves to reduce resistance during locomotion [24]. The wedge-shaped head of the sandfish aids in minimizing penetration resistance and maximizing propulsive force. Observation using high-speed cameras and infrared imaging has revealed that the sandfish exhibits two distinct lifestyles: aboveground and underground. When foraging for food, the sandfish employs its limbs to crawl on the surface, while it quickly burrows into the ground for protection or rest. The process of sandfish burrowing can be segmented into two phases: penetration into the soil and subsequent movement [21]. During soil entry, the head oscillates up and down while the limbs push against the ground to generate force. Once beneath the sand, the limbs are held close to the body, with propulsion achieved through lateral head movements and body twisting.

#### 3.1.1. Body Motion Curve Acquisition and Model Establishment of Sandfish

In this study, we sought to replicate the natural habitat of the sandfish and observe its burrowing behavior. We referred to the existing literature to derive the sandfish swing curve equation [23], as depicted in Figure 1b. As the sandfish navigates through the sand, its body moves in a sinusoidal pattern. Over time, the equation [23] describing this curve can be expressed as follows:

(1) $$y=A\;\sin\frac{2\pi }{\lambda }\left(x+v\times t\right)$$

While moving, the curve equation is in change. In the equation, *v* represents the forward speed, *t* denotes the time elapsed after entering the soil, *A* signifies the amplitude, *λ* stands for the wavelength, and the equation represents the body curve equation at a specific time. *A/λ* = 0.25 ± 0.07, with the average value of *A* being 0.18 ± 0.03 bl and the average value of *λ* being 0.7 ± 0.1 bl. To simplify the curve equation, the establishment of the body model disregards the phase, resulting in *v* × *t* = 0. Given that the body length of an adult lizard ranges from 15 to 20 cm, *A* = 30 mm, and *λ* = 120 mm, then the equation becomes:

(2) y=30 sin(x×9 rad)

The sandfish swing body model was constructed using CATIA V5R20, as depicted in Figure 1c. 

#### 3.1.2. Head Contour Acquisition and Model Establishment of Sandfish

**Figure 2:**
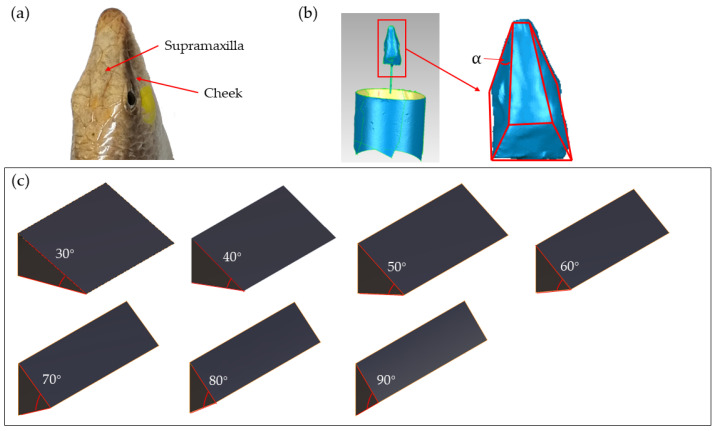
(**a**) Sandfish head; (**b**) head restoration model and angle measurement; (**c**) models with different angles.

As illustrated in Figure 2a, the head of the sandfish is characterized by a wedge shape with four distinct surfaces, and flat cheeks on either side. The upper and lower jaws form an angle denoted as α, with the lower jaw nearly parallel to the abdomen. In Figure 2b, the head was immobilized as a specimen, scanned using a three-dimensional scanner, and subsequently processed using reverse engineering techniques to generate a reconstructed three-dimensional model. The feature curve of the point cloud data was extracted through two steps using reverse engineering software (Imageware 13.2): Sharp Edges and Color-Based Point Cloud. The Sharp Edge function delineated the surface boundary of the point cloud, which was then saved and imported into AutoCAD 2020 for further decomposition into feature curve points. Subsequently, the corresponding coordinate values were exported and imported into Microsoft Office Excel 2016 for curve fitting. The key parameter of interest in this study pertains to the angle between the upper and lower jaws of the bionic sandfish. This angle was determined through curve analysis, yielding α = 27 ± 1°. The structural parameters of the model were finalized, as depicted in Figure 2c. Wedge models with angles of 30°, 40°, 50°, 60°, 70°, 80°, and 90° were constructed using CATIA V5R20, ensuring the area of each wedge facing the soil was consistent.

#### 3.1.3. Microstructure Model Establishment of Scale Surface

**Figure 3:**
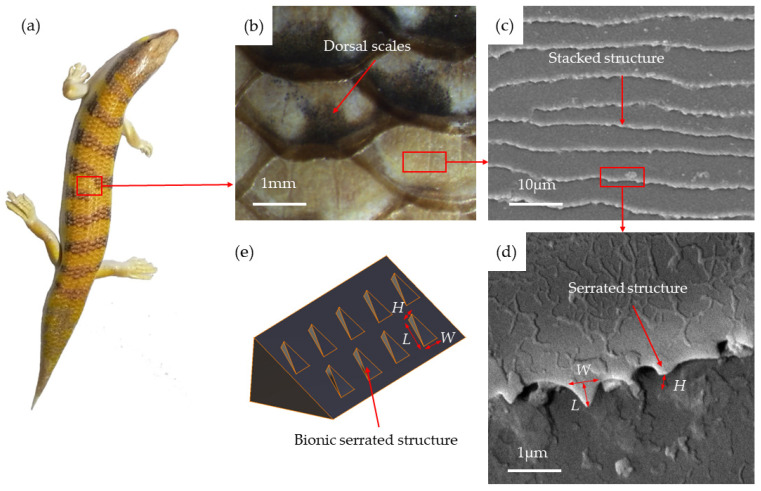
(**a**) Adult sandfish; (**b**) dorsal scales; (**c**) stripe structure of scale surface; (**d**) serrated structure of stripe end; (**e**) wedge model of bionic serrated structure.

The body surface of the sandfish exhibits a non-smooth microstructure. As illustrated in Figure 3, (a) depicts an adult sandfish, while (b) showcases the dorsal scales which are arranged in a superimposed manner. The wavy stripes on the scales surface, shown in (c), follow a similar arrangement to the scales. Additionally, (d) displays the triangular serrated structure located at the end of the stripe, which is slightly raised. The length *L*, width *W*, and height *H* of the serrated structure were observed and measured using an SEM. The relationship between these dimensions was determined to be *W/L* = 0.8–1.1 and *H/L* = 0.3–0.5. Based on these proportions, a bionic serrated structural wedge model was designed to mimic the serrated structure on the surface of the sandfish. The bionic serrated structural wedge model is displayed in (e).

### 3.2. Simulation of EDEM

#### 3.2.1. Soil Parameter Calibration

**Figure 4:**
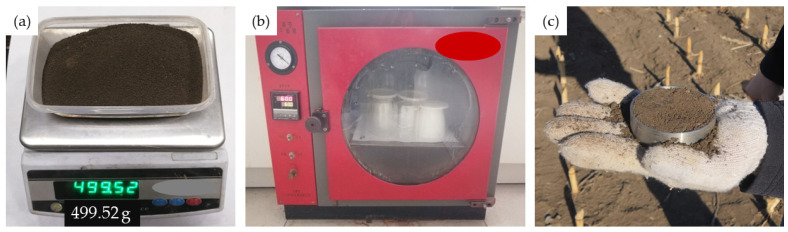
(**a**) Soil weighing; (**b**) soil drying; (**c**) soil sampling.

Given the potential applicability of soil anti-adhesion in agricultural machinery, we selected the local soil in Changchun City, Jilin Province, China to determine the relevant parameters. Figure 4 illustrates the measurement of basic soil parameters using the local field tillage layer soil. The soil moisture content on a dry basis was determined using the drying method [31], while the soil density was measured using the cutting ring method [32]. Figure 4 presents the measurement of soil mass (a), the process of drying soil (b), and the soil sampling using the cutting ring method (c). The calculation formula for soil dry basis moisture content is as follows:

(3) $$\omega_{0}=\left(\frac{m_{0}}{m_{d}}-\;1\right)\times 100\%$$

where *ω*_0_ is the soil moisture content, %; *m*_0_ is wet soil mass, kg; *m_d_* is the dry soil mass, kg.

The calculation formula for soil density is as follows:

(4) $$\rho =\frac{m}{V}$$

where *ρ* is the soil density, kg·m^−3^; *m* is soil mass, kg; *V* is the inner volume of the cutting ring, m^3^.

Through measurement and calculation, the soil’s dry-based moisture content is presented in Table 1, with an average value of 21.09%.

The variability in the soil composition within the field is significant, and due to the limited sampling capacity of the cutting ring, the average soil density measurement was utilized as a benchmark. The findings of the soil density measurements are presented in Table 2, indicating an average soil density of approximately 1663 kg·m^−3^.

**Figure 5:**
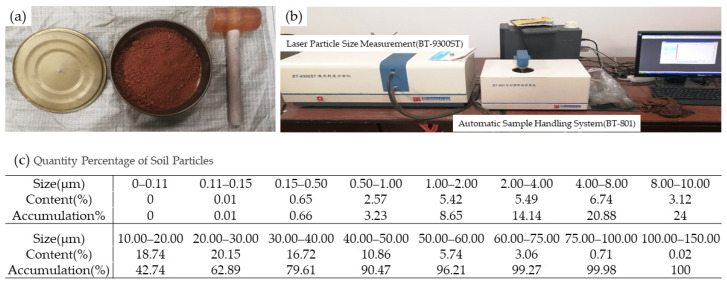
(**a**) Soil sieving; (**b**) soil analyzing; (**c**) results of soil analyzing.

The soil particle size distribution was determined using Laser Particle Size Measurement. After drying, the soil was sieved with a 2 mm analytical sieve to eliminate rhizomes, large stones, and other debris. The soil was then knocked and shattered to prepare the sample for analysis. Spare soil samples were collected for further analysis of soil texture using Laser Particle Size Measurement. The procedures and outcomes are presented in Figure 5a–c.

According to the international soil texture classification standard [33], soil particles are classified based on size into sand (0.02–2 mm), silt (0.002–0.02 mm), and clay (<0.002 mm). The mass percentage of the soil samples selected, categorized by sand, silt, and clay, is presented in Table 3 (Figure 5c). Based on this classification, the selected soil sample is identified as sandy loam.

**Figure 6:**
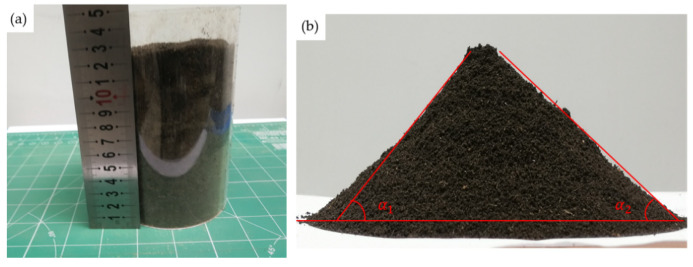
(**a**) Preparation of soil repose angle measurement; (**b**) process of soil repose angle measurement.

The soil accumulation test was conducted to determine the soil angle of repose [34]. In Figure 6, dried soil was used and prepared with a dry-based moisture content of 21%. A cylinder-type apparatus was selected to measure the soil angle of repose due to the stickiness of the wet soil. The prepared soil was thoroughly stirred to achieve a loose consistency before being placed in a bottomless cylinder. The cylinder was then removed vertically, allowing the soil to form different angles of accumulated mounds naturally on the loading platform. Once the soil mound reached stability, the inclination angle *α* was measured. The repose angle of sandy loam with a moisture content of 21% was determined to be 53° by averaging the left and right angles.

#### 3.2.2. Soil Parameter Calibration of EDEM

Because soil properties vary significantly, relevant soil parameters were utilized as independent variables. The material chosen for the soil-engaging component was 65 Mn steel. According to the literature, the Poisson ratio of 65 Mn steel is 0.3, with a density of 7850 kg·m^−3^, and a shear modulus of 7.9 × 10^10^ Pa [35]. Given that wet soil exhibits adhesion, the Hertz–Mindlin with JKR model [36] was selected as the physical contact model between particles, with surface energy as the independent variable. Soil adhesion is linked to soil texture, with soil clay content determining soil texture classification. Therefore, in the EDEM simulation process, the generation of soil particles must consider the influence of small particles. The particle model chosen was a single sphere with a calibrated radius of 1 mm. The particle size distribution was user-defined, taking into account the percentage of different types of soil particles in the sample. The simulated particle size distribution parameters were set as follows: small particles with a diameter of 1 mm accounted for 8.65% of the mass; medium particles with a diameter of 2 mm accounted for 34.09% of the mass; and large particles with a diameter of 3 mm accounted for 57.26% of the mass. To enhance simulation accuracy and efficiency, other parameter settings included a fixed time step of 10% in the simulator settings and a cell size equal to 3 times the radius of the particles. The specific factors and corresponding values are detailed in Table 4 [37,38].

Utilized Design-Expert 12 software to design the Plackett–Burman experimental design. Table 5 presents the experimental design scheme and simulation outcomes.

The significance of factors was analyzed in Table 6. The results indicate that factors *A*, *B*, *D*, and *E* had negative effects on the angle of repose, while factors *C*, *F*, *G*, *H*, *J*, and *K* had positive effects. Specifically, factors *D*, *E*, *F*, and *G* collectively accounted for 85.72% of the total contribution rate of significant factors.

The experiment on steepest climbing was conducted with four factors (*D*, *E*, *F*, *G*), while the levels of other factors were set at the median value. The results of the climbing tests were then compared with the measured soil repose angle of 53°. Analysis of Table 7 revealed that the relative error of Test Number 4 was 4.32%, indicating the closest approximation to the actual value.

Through the Plackett–Burman design and steepest climbing experiment, the factors in the climbing experiment were determined based on the Number 4 test, with the values of the Number 3 and Number 5 test factors set as the upper and lower limits, respectively. The range of values for the four factors was established, with the remaining factors set at the middle level. Subsequently, a regression equation and optimization analysis were conducted, and a Box–Behnken experimental design was implemented. Table 8 presents the Box–Behnken experimental design and results.

The test results were analyzed using a multiple regression equation to determine the regression equation for the soil repose angle, denoted as *Y*.

(5) $$\begin{array}{c} Y=-8.41-427.10\times D+12.93\times E+360.99\times F-13.85\times G-212.53\times DE\\ +276.32\times DF+815.26\times DG-102.10\times EF-7.00\times EG-222.75\times FG\\ +454.55\times D^{2}+37.10\times E^{2}-222.64\times F^{2}+10.23\times G^{2} \end{array}$$

The coefficient of determination for the regression equation was *R*^2^ = 0.9330, the adjusted coefficient $R_{adj}^{2}$ = 0.8661, the coefficient of variation *C.V.* = 6.88%, and the precision adequacy ratio *Adeq Precision* = 14.2648. In the analysis of variance for the quadratic polynomial model, the model’s *F*-value was 13.94 with a *p*-value of 0.0001, indicating a strong linear relationship. Consequently, the regression equation exhibited high accuracy and the model demonstrated excellent reliability. The angle of repose for soil with varying textures and moisture content varied significantly. Utilizing this regression equation to estimate the range of each factor based on different angles of repose can significantly reduce the workload associated with calibrating different soil simulations.

**Figure 7:**
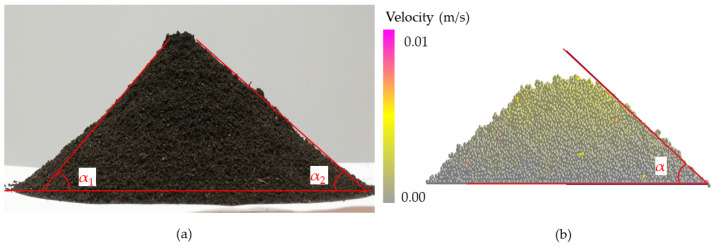
(**a**) Measurement of soil repose angle; (**b**) simulation of soil repose angle.

As shown in Figure 7a, the average repose angle of sandy loam with a moisture content of 21% was 53°. We selected the angle of 53° as the standard value to calibrate the regression equation and determine the target value for obtaining multiple sets of optimization values. We used the optimal combination and set other factors at the medium level for simulation verification. The simulation results, shown in Figure 7b, closely resembled the measured picture, indicating the superior reliability of the calibration results for this set of simulation parameters. The calibration results of EDEM simulation parameters for sandy loam with a moisture content of 21% are presented in Table 9.

#### 3.2.3. EDEM Simulation of Sandfish Body Swing

**Figure 8:**
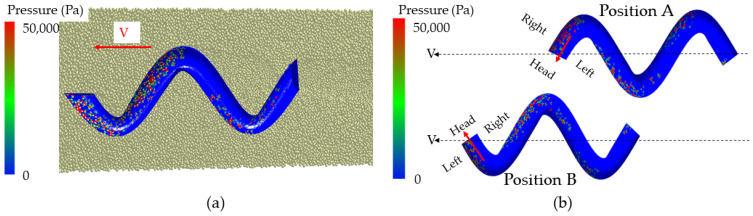
(**a**) EDEM simulation of the movement of sandfish model in soil; (**b**) states at different times of sandfish model.

In the process of underground movement, the sandfish’s head oscillates from left to right, causing its cheeks to make alternating contact with soil particles on both sides, resulting in a cyclic pressure that generates continuous power. Figure 8a illustrates the lizard body model’s movement within soil particles at a specific moment, with different colors on the model’s surface indicating varying pressures. The successive states of the body model transitioning from position A to position B are depicted in Figure 8b. It is evident from the illustration that the pressure on either side of the model fluctuated periodically, reflecting the fundamental principle of flexible desorption.

#### 3.2.4. Simulation of the Wedge Structure and Serrated Structure

**Figure 9:**
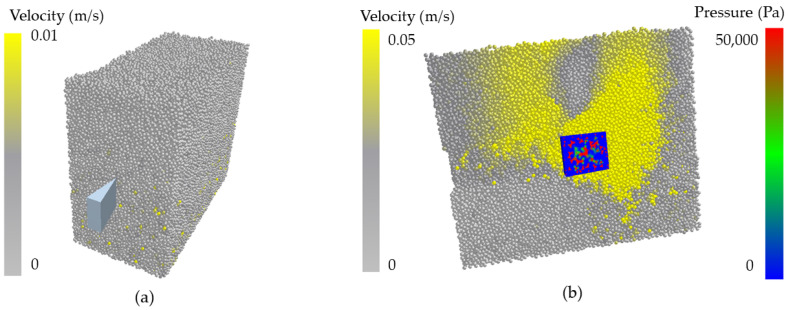
(**a**) Soil particle generation and wedge model import; (**b**) the state of wedges at a certain moment.

When simulating the motion of the wedge using EDEM software, stable and adequate soil particles were initially generated. Wedges with varying angles of 20°, 30°, 40°, 50°, 60°, 70°, 80°, and 90° were then imported to simulate their movement process, and the resistance of each wedge was analyzed. Figure 9 illustrates the soil particle generation and wedge model import in (a), while (b) shows the movement of the 20° wedge in the soil.

**Figure 10:**
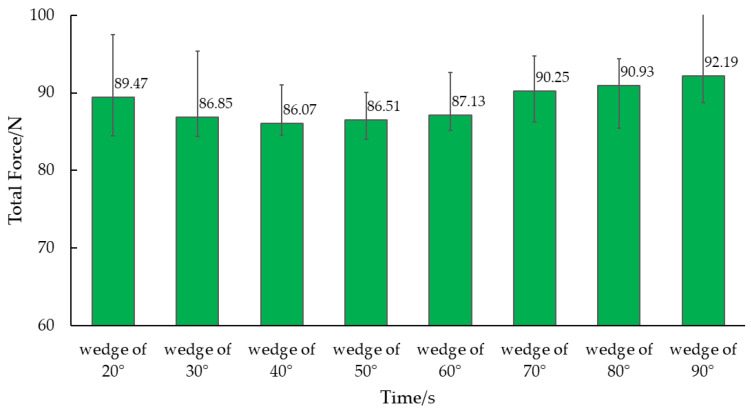
Simulate motion resistance of different wedges.

The motion resistance of wedges in the soil exhibits significant fluctuations. To determine the average value of motion resistance, a stable interval of 0.1–0.3 s was identified in the middle of the simulation time. Figure 10 illustrates the average resistance values for wedges at angles of 20°, 30°, 40°, 50°, 60°, 70°, 80°, and 90°, which were found to be 89.47 N, 86.85 N, 86.07 N, 86.51 N, 87.13 N, 90.25 N, 90.93 N, and 92.19 N, respectively. The figure reveals that the resistance of the 40° wedge was the smallest, similar to that of the 30° and 50° wedges, while the resistance of the 90° wedge was the largest.

**Figure 11:**
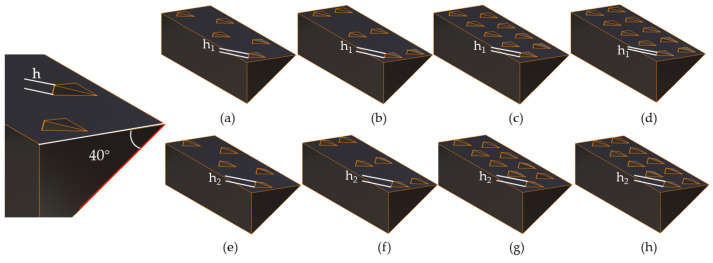
Non-smooth surface wedges with different microstructures: (**a**) wedge of low-tail sparse staggered distribution; (**b**) wedge of low-tail sparse parallel distribution; (**c**) wedge of low-tail dense staggered distribution; (**d**) wedge of low-tail dense parallel distribution; (**e**) wedge of high-tail sparse staggered distribution; (**f**) wedge of high-tail sparse parallel distribution; (**g**) wedge of high-tail dense staggered distribution; (**h**) wedge of high-tail dense parallel distribution.

The surface of the 40° wedge was designed with a non-smooth structure, imitating a scale surface serrated structure. The design also included a microstructure on the wedge surface. Three factors were considered in designing the non-smooth surface of the wedge to determine the best way to generate and arrange the serrated structure: the height of the serrated tail, arrangement mode, and arrangement density. Each factor had two levels, as shown in Table 10 in the simulation test factors and levels. Figure 11 illustrates eight types of bionic non-smooth surface wedges that were designed: (a) wedge of low-tail sparse staggered distribution, (b) wedge of low-tail sparse parallel distribution, (c) wedge of low-tail dense staggered distribution, (d) wedge of low-tail dense parallel distribution, (e) wedge of high-tail sparse staggered distribution, (f) wedge of high-tail sparse parallel distribution, (g) wedge of high-tail dense staggered distribution, and (h) wedge of high-tail dense parallel distribution.

**Figure 12:**
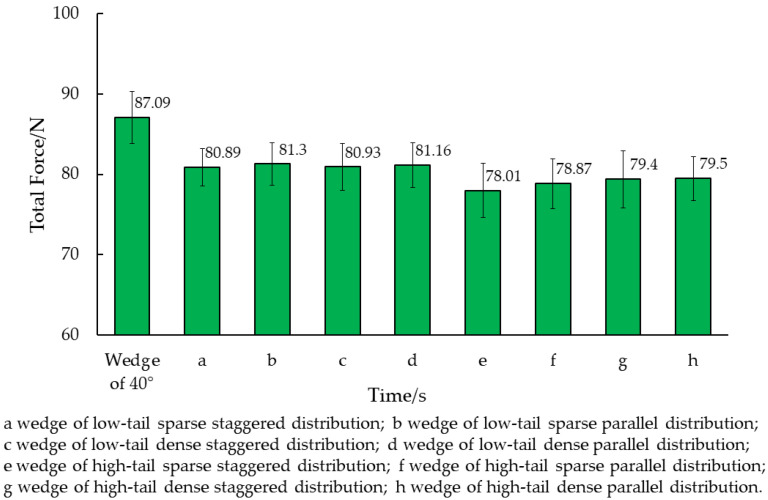
Simulation results of wedge resistance with different microstructures.

After conducting EDEM simulations, we determined the dynamic friction of wedges with various surface structures compared to smooth surfaces, all at a 40° angle. The average value of the stable interval was calculated for comparative analysis. As illustrated in Figure 12, the smooth surface wedge at 40° was compared to wedges with biomimetic non-smooth surfaces. The wedges were ranked based on Figure 11 as follows: (a) low-tail sparse staggered distribution, (b) low-tail sparse parallel distribution, (c) low-tail dense staggered distribution, (d) low-tail dense parallel distribution, (e) high-tail sparse staggered distribution, (f) high-tail sparse parallel distribution, (g) high-tail dense staggered distribution, and (h) high-tail dense parallel distribution.

The drag reduction percentages of the bionic structure wedge and eight different non-smooth surface serrated structure wedges were analyzed and compared, as shown in Table 11. The table reveals that the eight non-smooth surface serrated structure wedges exhibited varying degrees of drag reduction when compared to the smooth surface wedge. Among them, wedge (e) with a high-tail sparse staggered distribution serrated structure exhibited the highest drag reduction effect at 10.73%.

## 4. Discussion

### 4.1. Bionic Body Swing Desorption Mechanism of Sandfish

During the crawling process of reptiles, such as snakes and lizards, their bodies exhibit periodic reciprocating motion, which results in flexible anti-adhesion. As depicted in Figure 8, when the sandfish swings left and right, the pressure on both sides of its soil-engaging face changes periodically. When one side pushes against the soil, it generates a greater pressure, while the other side remains separated from the soil, resulting in lower pressure. As the sandfish’s body swings sinusoidally, the soil is divided into smaller segments. Each time it interacts with the soil to generate force, only a small portion of the area comes into contact with the soil, reducing the contact area. With the same applied force, the reduced contact area leads to significant periodic changes in surface pressure, facilitating the desorption effect. This principle resembles the motion mode of impact and vibration [39], which concentrates stress on the contact surface to achieve desorption.

### 4.2. The Interaction Mechanism between Bionic Sandfish Wedge and Soil

Non-smooth surfaces with bionic structures have been widely utilized in the field of bionics. The wedge-shaped head of the sandfish is divided into upper and lower jaws, as well as left and right buccal surfaces. The upper and lower jaws are wedge-shaped, with an angle of 27 ± 1°. The lateral cheeks are nearly perpendicular to the upper and lower jaws, with an angle of 80–90°. As the sandfish burrows into the sand, the smaller wedge angle created by the upper and lower jaws facilitates the slipping motion between the sand and the body surface, thereby reducing friction and aiding in rapid burrowing. During movement beneath the sand, the cheeks on both sides lie flat and are perpendicular to the sand surface. The larger angle at which the sand allows for greater force generation [40].

**Figure 13:**
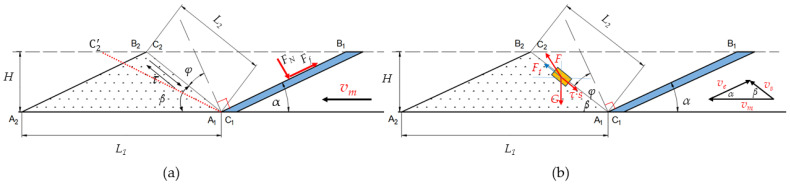
(**a**) Motion state of the wedge. (**b**) Motion state of the soil particle.

The soil conditions are complex, and the movement of soil particles is uncertain. Figure 13 illustrates the interaction between the soil-engaging components and the soil, showing that the wedge effect on the soil can be characterized by two processes: compaction and rupture. Specifically, this refers to the compaction and uplift of soil particles. The motion state of the wedge is depicted in Figure 13a. In an ideal scenario, when the wedge moves at a constant velocity *v_m_*, the wedge surface transitions from A_1_B_1_ to A_2_B_2_, causing soil particles to be lifted from point C_1_ to C_2_ due to the wedge action. The wedge angle of the two-sided wedge is denoted as *α*, the translation distance as *L*_1_, the effective depth of the wedge penetration into the soil as *H*, the movement distance of the soil particles as *L*_2_, the normal angle *φ* between points C_1_C_2_ and the wedge surface A_1_B_1_ as the external friction angle of the soil, and *τ* as the shear stress between the soil particles. In a simplified analysis that neglects adhesion forces, the soil’s force on the wedge can be primarily categorized into positive pressure and friction force. These forces are related as follows:

(6) $$F_{1}=F_{N}+F_{f}$$

where *F*_1_ is the resultant force of the contact surface, *F_N_* is the normal load of the contact surface, and *F_f_* is the tangential friction of the contact surface.

Assuming that the soil properties and the material of the soil-engaging components remain constant, the shear stress *τ* and the friction angle *φ* between the soil and the contact surface also remain constant even when the soil is disrupted. By applying Coulomb’s law of friction, the relationship shown in Figure 13 can be derived.

(7) $$F_{f}=\mu \times {\;F}_{N}$$

(8) μ=tan⁡φ

(9) α+β+φ=90°

where *μ* is the friction coefficient between the soil and the contact surface, *φ* is the friction angle between the soil and the contact surface, and *β* is the slip angle between the soil particles.

The motion state of the soil particle is illustrated in Figure 13b. The wedge moves uniformly at a horizontal speed *v_m_*, causing the soil particle to move along the soil slip surface at a speed *v_s_*. The relative velocity of the two is *v_e_*, and the angle with the horizontal plane is *α*, which is equal to the wedge angle. As the soil particle moves, it experiences its gravity *G*, the lifting force *F* provided by the wedge, and the shear stress *τ* between the soil particles along the slip surface. Assuming the soil particle moves at a constant speed, the three forces reach equilibrium, resulting in the following equation:

(10) F+G+τ×s=0

where *s* is the stressed area of the soil particle.

As the wedge angle *α* increases, the friction angle *φ* remains constant. Consequently, angle *β* decreases, causing soil particle C_1_ to move towards $C_{2}^{\prime }$ instead of C_2_. Consequently, soil particles must traverse a greater distance, necessitating a stronger force for accelerated motion. Thus, based on theoretical analysis, a smaller wedge angle *α* corresponds to reduced soil resistance. Nonetheless, sandy loam soil with a 21% moisture content exhibits adhesion. With a very small wedge angle *α* and constant soil surface height *H*, there is a significant increase in the contact surface area between the soil and the wedge, resulting in heightened soil adhesion. Thus, in varying soil conditions, an optimal solution exists to minimize the motion resistance of the wedge. Figure 2, Figure 9 and Figure 10 depict simulations conducted on local sandy loam, demonstrating that the motion resistance was minimized when the wedge angle on both sides was 40°. 

### 4.3. The Anti-Adhesion Mechanism of Bionic Serrated Structure

The swinging motion during reptile crawling serves as an anti-adhesion mechanism, similar to the effects of bristles. In the presence of a normal force at the interface between cohesive soil and a solid surface, the sliding resistance in the low-stress region is minimal under equal stress conditions. As the normal force intensifies, the sliding resistance between the interface and the soil escalates rapidly [41,42].

**Figure 14:**
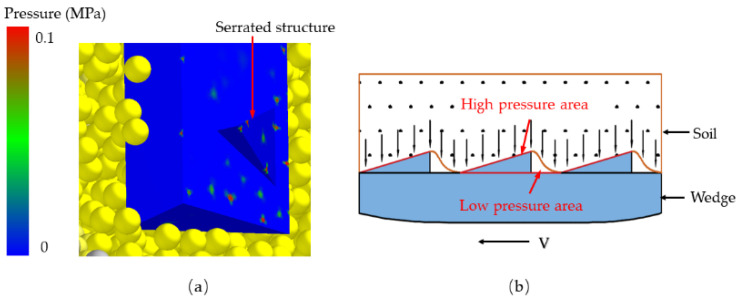
(**a**) The stress distribution on the surface of the wedge; (**b**) the contact model of soil and soil-engaging components.

The morphology of soil-engaging components significantly influences soil movement. Soil particles with adhesive properties tend to clump together, forming a cohesive mass during transportation. Throughout the motion process, the soil-engaging components interact with the soil as a unified entity. Figure 14a illustrates that the tail surface of the serrated structure experiences higher pressure, creating a low-pressure zone around the structure. In contrast, Figure 14b demonstrates how the serrated structure partitions the soil into distinct segments, exhibiting a stronger interaction force with the soil. This results in the formation of alternating high-pressure and low-pressure zones, resembling the movement pattern of a mollusk. Hence, the serrated structure facilitates soil desorption.

In this study, a bionic serrated structure was utilized to create a non-smooth surface wedge model to simulate its penetration resistance. The findings are depicted in Figure 12. Among the wedges featuring various bionic structures, the high-tail sparse staggered distributed structure exhibited the lowest simulated resistance. When compared to the smooth surface wedge, it demonstrated the most effective drag reduction at approximately 10.73%. Nevertheless, the limited number of models and factor variable intervals in this simulation necessitate further investigation into specific values such as the height of the serrated end and distribution densities.

## 5. Conclusions

In the process of burrowing under the sand, the sandfish’s body swings periodically, leading to changes in pressure on its surface. This can result in an effective desorption effect, demonstrating the efficiency of flexible desorption. The wedge-shaped head structure of the sandfish not only generates sufficient forward force but also facilitates soil penetration. Additionally, the serrated surface structure of the scales creates a periodic variation in pressure when in contact with the soil, further confirming the efficacy of flexible drag reduction.

The interaction between different types of soil and soil-engaging components varies significantly. When using EDEM, it is necessary to calibrate the relevant parameters of both the soil and the components. In this study, soil samples from Changchun City, Jilin Province, China were selected, with a moisture content of 21%. The simulation parameters were calibrated based on the soil repose angle, resulting in highly consistent simulated values with the measured data.

Based on the calibrated soil parameters, simulations were conducted to analyze the wedge-shaped structure of the sandfish’s head. The results showed that a 40° wedge experienced the least resistance when moving through sandy loam. By mimicking the flexible desorption mechanism of the sandfish, a serrated structure was added to the surface of the soil-engaging component, leading to effective drag reduction. Specifically, a wedge with a high serrated tail, sparse distribution, and staggered arrangement exhibited the best drag reduction performance, and the drag reduction was about 10.73%.

## Figures and Tables

**Table 1:** Soil moisture content measurement results.

Physical Quantity	Group 1	Group 2	Group 3
$m_{0}$ (g)	499.52	499.48	500.32
$m_{d}\;$(g)	410.56	415.12	412.52
$\omega_{0}\;$(%)	21.67	20.32	21.28

**Table 2:** Soil density measurement results.

Physical Quantity	Group 1	Group 2	Group 3
m (kg)	0.153	0.172	0.164
v (m^3^)	9.8 × 10^−5^	9.8 × 10^−5^	9.8 × 10^−5^
ρ (kg·m^−3^)	1561	1755	1673

**Table 3:** Soil particle content of different grades.

Soil Particle	Sand (0.02–2 mm)	Silt (0.002–0.02 mm)	Clay (<0.002 mm)
Mass percentage (%)	57.26	34.09	8.65

**Table 4:** Simulation of soil factors and level setting by EDEM.

EDEM Parameters	Factor		Level		
			−1	0	1
Bulk material	Poisson ratio	*A*	0.3	0.4	0.5
	Solids density (kg·m^−3^)	*B*	1600	2100	2600
	Shear modulus (MPa)	*C*	10	20	30
Particle–particle interaction	Coefficient of restitution	*D*	0.01	0.2	0.4
	Coefficient of static friction	*E*	0.2	0.7	1.2
	Coefficient of rolling friction	*F*	0.1	0.2	0.7
Particle–steel interaction	Coefficient of restitution	*G*	0.01	0.2	0.4
	Coefficient of static friction	*H*	0.2	0.7	1.2
	Coefficient of rolling friction	*J*	0.1	0.3	0.7
Physical interaction model	Hertz–Mindlin with JKR (J·m^−2^)	*K*	0	2	4

**Table 5:** Plackett–Burman design and simulation results.

Run	Factor											Response (°)
	*A*	*B*	*C*	*D*	*E*	*F*	*G*	*H*	*J*	*K*	*L*	
1	0.5	2600	30	0.01	0.2	0.1	0.4	0.2	0.7	4	−1	32.65
2	0.5	2600	10	0.4	1.2	0.7	0.01	0.2	0.1	4	−1	31.8
3	0.5	1600	10	0.01	1.2	0.1	0.4	1.2	0.1	4	1	32.36
4	0.3	1600	10	0.4	0.2	0.7	0.4	0.2	0.7	4	1	56.36
5	0.3	2600	30	0.01	1.2	0.7	0.4	0.2	0.1	0	1	46.63
6	0.3	2600	10	0.4	1.2	0.1	0.4	1.2	0.7	0	−1	25.9
7	0.5	1600	30	0.4	1.2	0.1	0.01	0.2	0.7	0	1	23.37
8	0.3	1600	10	0.01	0.2	0.1	0.01	0.2	0.1	0	−1	22.36
9	0.5	1600	30	0.4	0.2	0.7	0.4	1.2	0.1	0	−1	45.16
10	0.5	2600	10	0.01	0.2	0.7	0.01	1.2	0.7	0	1	54.52
11	0.3	2600	30	0.4	0.2	0.1	0.01	1.2	0.1	4	1	27.47
12	0.3	1600	30	0.01	1.2	0.7	0.01	1.2	0.7	4	−1	50.61

**Table 6:** Results of significance analysis of experimental factors.

Factor	Standardized Effect	Sum of Squares	Contribution	Significance
*A*-*A*	−1.58	7.49	1.25	8
*B*-*B*	−1.88	10.57	1.76	7
*C*-*C*	0.43	0.5547	0.0924	11
*D*-*D*	−4.84	70.37	11.72	2
*E*-*E*	−4.64	64.68	10.77	4
*F*-*F*	10.16	309.88	51.61	1
*G*-*G*	4.82	69.79	11.62	3
*H*-*H*	3.81	43.47	7.24	5
*J*-*J*	1.27	4.86	0.8101	9
*K*-*K*	2.22	14.79	2.46	6
*AB*	−5.37	4	0.6665	10

**Table 7:** Test scheme and results of the steepest climbing experiment.

Run	Significant Factors				Response (°)	Relative Error (%)
	*D*	*E*	*F*	*G*		
1	0.4	1.2	0.1	0.01	21.16	60.08%
2	0.3	0.95	0.25	0.1	31.11	40.87%
3	0.2	0.7	0.4	0.2	42.67	19.49%
4	0.1	0.45	0.55	0.3	50.76	4.23%
5	0.01	0.2	0.7	0.4	62.55	18.02%

**Table 8:** Test scheme and results of the Box–Behnken experimental design.

Run	Factor				Response (°)
	*D*	*E*	*F*	*G*	
1	0.105	0.7	0.55	0.4	45.52
2	0.105	0.45	0.55	0.3	48.54
3	0.01	0.2	0.55	0.3	62.37
4	0.2	0.45	0.55	0.4	53.39
5	0.105	0.2	0.55	0.4	55.94
6	0.105	0.2	0.4	0.3	47.9
7	0.2	0.45	0.7	0.3	61.38
8	0.2	0.45	0.55	0.2	42.46
9	0.2	0.7	0.55	0.3	45.4
10	0.2	0.2	0.55	0.3	59.94
11	0.2	0.45	0.4	0.3	31.18
12	0.105	0.45	0.55	0.3	52.23
13	0.01	0.45	0.55	0.4	55.95
14	0.105	0.45	0.4	0.4	44.27
15	0.105	0.45	0.7	0.2	60.94
16	0.105	0.45	0.55	0.3	52.15
17	0.105	0.7	0.7	0.3	52.68
18	0.105	0.2	0.55	0.2	58.34
19	0.105	0.7	0.55	0.2	48.62
20	0.105	0.45	0.55	0.3	51.82
21	0.105	0.45	0.4	0.2	41.12
22	0.01	0.45	0.4	0.3	46.97
23	0.01	0.45	0.7	0.3	66.67
24	0.105	0.45	0.7	0.4	55.18
25	0.01	0.45	0.55	0.2	76.03
26	0.01	0.7	0.55	0.3	68.02
27	0.105	0.2	0.7	0.3	71.68
28	0.105	0.7	0.4	0.3	39.11
29	0.105	0.45	0.55	0.3	53.52

**Table 9:** Results of the EDEM simulation parameter calibration.

EDEM Simulation Parameters	Factor	Value
Sandy loam soil	Poisson ratio	0.4
	Solids density (kg·m^−3^)	2100
	Shear modulus (MPa)	20
65 Mn steel	Poisson ratio	0.3
	Solids density (kg·m^−3^)	7850
	Shear modulus (MPa)	7.9 × 10^4^
Particle–particle interaction	Coefficient of restitution	0.187
	Coefficient of static friction	0.54
	Coefficient of rolling friction	0.462
Particle–steel interaction	Coefficient of restitution	0.355
	Coefficient of static friction	0.7
	Coefficient of rolling friction	0.5
Physical interaction model	Hertz–Mindlin with JKR (J·m^−2^)	4

**Table 10:** Simulation factors of wedges with different microstructures.

Factor	Level	
	−1	1
Distribution method	parallel	staggered
Distribution density	sparse	dense
Serrated tail height	low	high

**Table 11:** The motion resistance and drag reduction percentage of wedges with different structures.

Wedges	Wedge of 40°	a	b	c	d	e	f	g	h
Motion Resistance	87.09	80.89	81.30	80.93	81.16	78.01	78.87	79.40	79.50
Drag Reduction Percentage		7.12%	6.65%	7.07%	6.81%	10.73%	9.44%	8.83%	8.72%

## Data Availability

The data presented in this study are available on request from the corresponding author.

## References

[1] Merkisz J., Lijewski P., Fuc P., Siedlecki M., Weymann S. (2015). The Use of the PEMS Equipment for the Assessment of Farm Fieldwork Energy Consumption. Appl. Eng. Agric..

[2] Scarpare F.V., de Jong van Lier Q., de Camargo L., Pires R.C.M., Ruiz-Corrêa S.T., Bezerra A.H.F., Gava G.J.C., Dias C.T.S. (2019). Tillage Effects on Soil Physical Condition and Root Growth Associated with Sugarcane Water Availability. Soil. Tillage Res..

[3] Zhou D., Hou P., Xin Y., Wu B., Tong J., Yu H., Qi J., Zhang J., Zhang Q. (2021). Resistance and Consumption Reduction Mechanism of Bionic Vibration and Verification of Field Subsoiling Experiment. Appl. Sci..

[4] Wang Y., N Osman A., Zhang D., Yang L., Cui T., Zhong X. (2019). Optimized Design and Field Experiment of a Staggered Vibrating Subsoiler for Conservation Tillage. Int. J. Agric. Biol. Eng..

[5] Mehrang Marani S., Shahgholi G., Moinfar A. (2019). Effect of Nano Coating Materials on Reduction of Soil Adhesion and External Friction. Soil. Tillage Res..

[6] Wang Y., Zhang D., Yang L., Cui T., Jing H., Zhong X. (2020). Modeling the Interaction of Soil and a Vibrating Subsoiler Using the Discrete Element Method. Comput. Electron. Agric..

[7] Zhang X.R., Wang C., Chen Z.H., Zeng Z.W. (2016). Design and Experiment of a Bionic Vibratory Subsoiler for Banana Fields in Southern China. Int. J. Agric. Biol. Eng..

[8] Massah J., Rahmani Fard M., Aghel H. (2021). An Optimized Bionic Electro-Osmotic Soil-Engaging Implement for Soil Adhesion Reduction. J. Terramech.

[9] Li J., Tong J., Ren L., Chen B. Applications of Bionics Non-Smooth Surface to Reduce Draft Resistance against Soil. Proceedings of the 14th International Conference of the Society for Terrain-Vehicle Systems.

[10] Ren L., Wang S., Tian X., Han Z., Yan L., Qiu Z. (2007). Non-Smooth Morphologies of Typical Plant Leaf Surfaces and Their Anti-Adhesion Effects. J. Bionic Eng..

[11] Li J., Tong J., Hu B., Ma Y. (2019). Biomimetic Functional Surface of Reducing Soil Adhesion on 65Mn Steel. Adv. Mech. Eng..

[12] Zhang Y., Zhou C., Ren L. (2008). Biology Coupling Characteristics of Mole Crickets’ Soil-Engaging Components. J. Bionic Eng..

[13] Grützmacher P.G., Profito F.J., Rosenkranz A. (2019). Multi-Scale Surface Texturing in Tribology-Current Knowledge and Future Perspectives. Lubricants.

[14] Hsu S.M., Jing Y., Zhao F. (2016). Self-Adaptive Surface Texture Design for Friction Reduction across the Lubrication Regimes. Surf. Topogr..

[15] Schille J., Schneider L., Mauersberger S., Szokup S., Höhn S., Pötschke J., Reiß F., Leidich E., Löschner U. (2020). High-Rate Laser Surface Texturing for Advanced Tribological Functionality. Lubricants.

[16] Costa H.L., Schille J., Rosenkranz A. (2022). Tailored Surface Textures to Increase Friction—A Review. Friction.

[17] Ren L. (2009). Progress in the Bionic Study on Anti-Adhesion and Resistance Reduction of Terrain Machines. Sci. China.

[18] Li M., Chen D., Zhang S., Tong J. (2013). Biomimeitc Design of a Stubble-Cutting Disc Using Finite Element Analysis. J. Bionic Eng..

[19] Xu S., Wang C., Mao R., Liang X., Wang H., Lin Z., Li J., Li S., Jiang J., Zhang T. (2022). Surface Structure Change Properties: Auto-Soft Bionic Fibrous Membrane in Reducing Postoperative Adhesion. Bioact. Mater..

[20] Luo X., Dong X., Zhao H., Hu T.S., Lan X., Ding L., Li J., Ni H., Contreras J.A., Zeng H. (2022). Near-Infrared Responsive Gecko-Inspired Flexible Arm Gripper. Mater. Today Phys..

[21] Ding Y., Li C., Goldman D.I. (2013). Swimming in the Desert. Phys. Today.

[22] Maladen R.D., Ding Y., Umbanhowar P.B., Kamor A., Goldman D.I. (2011). Mechanical Models of Sandfish Locomotion Reveal Principles of High Performance Subsurface Sand-Swimming. J. R. Soc. Interface.

[23] Ding Y., Sharpe S.S., Masse A., Goldman D.I. (2012). Mechanics of Undulatory Swimming in a Frictional Fluid. PLoS Comput. Biol..

[24] Vihar B., Hanisch F.G., Baumgartner W. (2016). Neutral Glycans from Sandfish Skin Can Reduce Friction of Polymers. J. R. Soc. Interface.

[25] Baumgartner W., Saxe F., Weth A., Hajas D., Sigumonrong D., Emmerlich J., Singheiser M., Bohme W., Schneider J.M. (2007). The Sandfish’s Skin: Morphology, Chemistry and Reconstruction. J. Bionic Eng..

[26] Wu W., Lutz C., Mersch S., Thelen R., Greiner C., Gomard G., Hölscher H. (2018). Characterization of the Microscopic Tribological Properties of Sandfish (*Scincus scincus*) Scales by Atomic Force Microscopy. Beilstein J. Nanotechnol..

[27] Staudt K., Böhme W., Baumgartner W. (2012). Comparative Investigations of the Sandfish’s β-Keratin (Reptilia: Scincidae: *Scincus Scincus*). Part 2: Glycan-Based Friction Reduction. J. Biomim. Biomater. Tissue Eng..

[28] Staudt K., Saxe F.P.M., Schmied H., Soeur R., Böhme W., Baumgartner W. (2012). Comparative Investigations of the Sandfish’s β-Keratin (Reptilia: Scincidae: *Scincus scincus*). Part 1: Surface and Molecular Examinations. J. Biomim. Biomater. Tissue Eng..

[29] Goldman D.I. (2014). *Colloquium*: Biophysical Principles of Undulatory Self-Propulsion in Granular Media. Rev. Mod. Phys..

[30] Maladen R.D., Ding Y., Umbanhowar P.B., Goldman D.I. (2011). Undulatory Swimming in Sand: Experimental and Simulation Studies of a Robotic Sandfish. Int. J. Rob. Res..

[31] O’Kelly B.C. (2004). Accurate Determination of Moisture Content of Organic Soils Using the Oven Drying Method. Dry. Technol..

[32] Qiao Y., Zhu H., Zhong H., Li Y. (2020). Stratified Data Reconstruction and Spatial Pattern Analyses of Soil Bulk Density in the Northern Grasslands of China. ISPRS Int. J. Geoinf..

[33] Murano H., Takata Y., Isoi T. (2015). Origin of the Soil Texture Classification System Used in Japan. Soil. Sci. Plant Nutr..

[34] Yang Q., Shi L., Shi A., He M., Zhao X., Zhang L., Addy M. (2023). Determination of Key Soil Characteristic Parameters Using Angle of Repose and Direct Shear Stress Test. Int. J. Agric. Biol. Eng..

[35] Huang G., Jiao G.H., Wen D.Z., Zhou C.Q., Wu K.M. (2010). Mechanical Properties of Heat Treated 65Mn Steel Produced by Compact Strip Production. Adv. Mat. Res..

[36] Chen J., Krengel D., Nishiura D., Furuichi M., Matuttis H.-G. (2023). A Force–Displacement Relation Based on the JKR Theory for DEM Simulations of Adhesive Particles. Powder Technol..

[37] Shaolong S., Zhihui T., Xuan Z., Jinbao L., Xiangjin M., Yuchao L. (2021). Calibration of the Discrete Element Parameters for the Soil Model of Cotton Field after Plowing in Xinjiang of China. Trans. Chin. Soc. Agric. Eng..

[38] Huang S., Lu C., Li H., He J., Wang Q., Yuan P., Xu J., Jiang S., He D. (2023). Calibration of Acoustic-Soil Discrete Element Model and Analysis of Influencing Factors on Accuracy. Remote Sens..

[39] Zhao J.Z., Pan G., Gao S. (2020). Research on the Mechanism of Drag Reduction by Wall Vibration through Lattice Boltzmann Method. Mod. Phys. Lett. B.

[40] Koolen A.J., Kuipers H. (1983). Agricultural Soil Mechanics.

[41] Li J., Li Y., Zhang Z. (2004). Characteristics of Sliding Resistance Reduction between Soil and Flexible Body Surface of Yellow Mouse’s Living Body. Trans. Chin. Soc. Agric. Mach..

[42] Sun J., Li J., Cheng H., Dai Z., Rer L. (2004). Restudies on Body Surface of Dung Beetle and Application of Us Bionics Flexible Technique. J. Bionics Eng..

